# A population-based approach for implementing change from opt-out to opt-in research permissions

**DOI:** 10.1371/journal.pone.0168223

**Published:** 2017-04-25

**Authors:** Elizabeth A. Marshall, Jim C. Oates, Azza Shoaibi, Jihad S. Obeid, Melissa L. Habrat, Robert W. Warren, Kathleen T. Brady, Leslie A. Lenert

**Affiliations:** 1 Biomedical Informatics Center, Medical University of South Carolina, Charleston, South Carolina, United States of America; 2 Department of Public Health Sciences, Medical University of South Carolina, Charleston, South Carolina, United States of America; 3 Department of Medicine, Division of Rheumatology and Immunology, Medical University of South Carolina, Charleston, South Carolina, United States of America; 4 Medical Service, Rheumatology Section, Ralph H. Johnson VA Medical Center, Charleston, South Carolina, United States of America; 5 Department of Pediatrics, Division of Pediatric Rheumatology and Immunology, Medical University of South Carolina, Charleston, South Carolina, United States of America; 6 Department of Psychiatry and Behavioral Sciences, Medical University of South Carolina, Charleston, South Carolina, United States of America; 7 Department of Medicine, Division of General Internal Medicine, Medical University of South Carolina, Charleston, South Carolina, United States of America; Stellenbosch University Faculty of Medicine and Health Sciences, SOUTH AFRICA

## Abstract

Due to recently proposed changes in the Common Rule regarding the collection of research preferences, there is an increased need for efficient methods to document opt-in research preferences at a population level. Previously, our institution developed an opt-out paper-based workflow that could not be utilized for research in a scalable fashion. This project was designed to demonstrate the feasibility of implementing an electronic health record (EHR)-based active opt-in research preferences program. The first phase of implementation required creating and disseminating a patient questionnaire through the EHR portal to populate discreet fields within the EHR indicating patients’ preferences for future research study contact (contact) and their willingness to allow anonymised use of excess tissue and fluid specimens (biobank). In the second phase, the questionnaire was presented within a clinic nurse intake workflow in an obstetrical clinic. These permissions were tabulated in registries for use by investigators for feasibility studies and recruitment. The registry was also used for research patient contact management using a new EHR encounter type to differentiate research from clinical encounters. The research permissions questionnaire was sent to 59,670 patients via the EHR portal. Within four months, 21,814 responses (75% willing to participate in biobanking, and 72% willing to be contacted for future research) were received. Each response was recorded within a patient portal encounter to enable longitudinal analysis of responses. We obtained a significantly lower positive response from the 264 females who completed the questionnaire in the obstetrical clinic (55% volunteers for biobank and 52% for contact). We demonstrate that it is possible to establish a research permissions registry using the EHR portal and clinic-based workflows. This patient-centric, population-based, opt-in approach documents preferences in the EHR, allowing linkage of these preferences to health record information.

## Introduction

At the Medical University of South Carolina (MUSC), a five-year strategic plan was recently implemented with one goal to “commit to patients first” [1]. Congruent with this goal, we describe the approach for changing our legacy opt-out paper-based method for capture of research permissions to an opt-in approach implemented within our Electronic Patient Record (EHR) system.

It is recognized that patients prefer to be *asked* about potential participation in research and use of fluids and tissues originating from the care in research [2,3]. Proposed revisions to research guidelines of the Office for Human Research Protections (OHRP) include requirements for broad consent for secondary research on surplus biospecimens [4]. While it is more patient-centric to ask patients for permission to use excess biospecimens, the question that many have raised is whether obtaining opt-in “broad consent” for use of surplus tissues is practical [5,6].

To address these new guidelines [7, 8], MUSC has moved from a paper-based passive opt-in (active opt-out) policy to an opt-in, electronic, population-based approach for gathering permissions for research contact and surplus specimen use. We chose to solicit research permissions from our clinical population within existing EHR clinical workflows [9–11]. The goal of this process was to overcome the inherent limitations of a paper-based passive opt-in approach by giving patients full control over their research permissions. One barrier to recruitment is the inability to rapidly identify willing, qualified participants. We believed that use of an EHR-integrated portal would enable us to 1) obtain and maintain permission for research contact on a large sample of the population within a secure EHR, 2) obtain preliminary qualification for specific research from their clinical record data, 3) use of the patient portal for direct recruitment or biospecimen collection, and 4) use of the patient portal and clinical workflows to change research permissions at any time. The purpose of this manuscript is to describe the methods and outcome of our approach to transitioning from a passive opt-in (active opt-out) to an active opt-in research permissions program integrated with the EHR. The goal of the project was to provide investigators with a registry of patients in the medical record who have given permission to be contacted about research or provide surplus tissue. This would allow investigators to use the EHR-based reporting and query tools for feasibility and recruitment.

### Historical perspective

**Fig 1:**
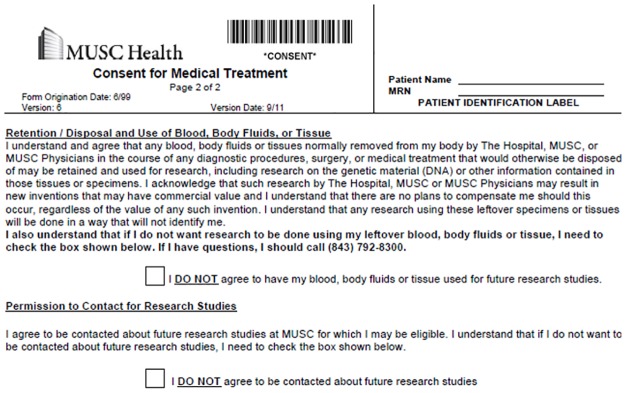
Active opt-out paper form.

In 2011, after a 2-year process of development, MUSC began collecting opt-out permission for direct contact for research and for use of surplus tissue for research. The approach was modeled on that taken in the creation of Vanderbilt’s BioVu repository [12]. Permissions were imbedded within MUSC’s Consent for Medical Treatment document presented at registration for encounters (Fig 1). Institutions have historically separated permission for research contact [13–19] and for surplus tissue use [20–24]. To improve efficiency, we sought to capture both types of permissions in a single, integrated, process. These paper-based forms were electronically scanned and uploaded to the patient’s medical record but were not available for rapid retrieval or reporting. In 2012–13, the institution conducted an initial pilot of capture of research permissions using a third-party registration application. This error rate for transcribing the paper-based permissions into the database was high (close to 14% by the end of the pilot), but the study demonstrated the clinical feasibility of using electronic data capture for research permissions [25–27].

### Toward a more patient-centric approach

In 2012, MUSC implemented a new EHR system (Epic). As part of this transition, the leadership of the South Carolina Translational Research Institute (SCTR) worked with the Biomedical Informatics Center (BMIC), SCTR’s research informatics group, and key hospital and University leadership to design and implement a new method of collecting research permissions within the EHR. A task force was created that included leadership from multiple stakeholders: SCTR, BMIC, the Institutional Review Board (IRB), the compliance offices, University legal counsel, the SCTR Community Engagement Core, the Department of Medicine, the Associate Provost’s office, community advisory boards, and the Chief Medical Information Officer (CMIO). The task force determined that patients should be given the ability to actively opt-in or opt-out. A third option was recommended: that patients be able to respond that they were “not ready to make a decision” as well. The task force acknowledged that while an EHR-based research permissions process could accelerate identification and recruitment of patients into clinical studies, its inherent efficiency could also unduly burden patients with multiple contacts for recruitment. Therefore, a contact management process was mandated that included auditing the number of contacts with individual patients.

## Methods

### Design parameters

In the design of a research permissions capture system for our EHR (Epic), we envisioned a comprehensive *patient-centric* process that would give each patient the opportunity declare their preferences regarding to direct contact for research and for use of their surplus tissues in research. A multi-modal process was envisioned, with patients completing a questionnaire either using the patient portal or in the outpatient clinic workflow. In clinic, consent would take place where the patient had time to contemplate the implications of their answers, privacy to respond however they wished, and access to a health professional to answer questions. As a result, we removed from consideration capturing preferences for research at the registration desk. In addition, a primary design consideration was that patients be able to access and change their research preferences at any time. This directed us toward making research preferences available to patients through the EHR patient portal. The language used in consent in the research permissions questionnaire duplicated the prior, approved opt-out paper form with two exceptions included the addition of a third response option as follows: 1) three response options were given to each question: “agree”, “disagree”, and “not ready to make a decision” and 2) formatting changes were required to implement the questionnaire within our Epic EHR.

### EHR data model modifications

New data elements were created to store separate research contact and tissue use permissions values within the record. These element values were set to: “agree (opt-in)”, “disagree (opt-out)”, and “not-ready-to-decide”. This third value was included to reduce a sense of coercion to respond. The values associated with these elements were stored in as a custom data element (Epic Smart Data Element) in Epic concept value (HLV) master file. Patient portal encounter (EHR classification of where/how encounters with patients occur) types specific to research were created to simplify contact management, which used foundation system population health management processes (reminder messages, alerts within the record, portal questionnaires) to maintain permissions. The research permissions attributes could change from encounter to encounter and were recorded at the level of the encounter. Changes to the questionnaire, the process of maintaining research permissions in the EHR, the contact management process, and the auditing functionality were reviewed and approved by the aforementioned task force and our Institutional Review Board.

### Use of the EHR for pre-research and cohort identification

To leverage the power of maintaining a research permissions registry within the EHR, we provided researchers with tools for both self-service and honest broker access to data in our local instance of Epic. Self-service queries using the Epic SlicerDicer (an Epic Cogito service) application were enabled by inclusion of two registries as potential inclusion criteria: 1) patients who had opted in for future research contact and 2) patients who had opted in for anonymised use of surplus biospecimens. Using SlicerDicer, a researcher can perform Boolean queries on any combination of coded data in the EHR linked by logical expressions to define patient cohorts including but not limited to: Systematized Nomenclature of Medicine (SNOMED) codes and groupers, problem list diagnoses, medications and doses, laboratory tests and ranges, vital signs, encounter type, demographics (with age ranges), registries (including the research registries), procedures, allergies, immunizations, and smoking status. These queries provide only aggregate data at a population level, but the query logic can be saved and shared with an honest broker, who can provide lists of patients meeting IRB-approved inclusion criteria. An additional mechanism available to researchers was to go through an IRB-approved honest broker to access Epic Reporting Workbench tools. These reports have preset inclusion logic and display fields that can be used for future recruitment queries [28]. Researchers can also obtain Epic InBasket (a provider work queue) notifications when patients meet specified combined clinical and research permissions registry criteria. This option is useful for timely recruitment or collection of surplus tissue in clinical laboratory.

### Research permissions registries

To manage use of research permissions for recruitment, we created a dedicated research permissions registries within the EHR that contains patients’ preferences for research as well as basic demographic data. The general research permissions registry included all patients who answered the research preferences questions. This general permissions registry was designed to aid contact management. Two additional registries were created: 1) a contact registry that contained patients who opted in for future research contact, and 2) a biobank registry that contained patients who had opted in for use of excess biospecimens. The latter two registries were created for inclusion rules for recruiting and feasibility queries. All registries are updated daily. The research permissions registry was designed to support several functions: 1) tracking which patients have and have not completed research preferences questionnaires; 2) managing annual renewal of permissions; 3) tracking and distributing information to patients who request more information; and 4) tracking research recruitment contact efforts.

### Contact management

**Fig 2:**
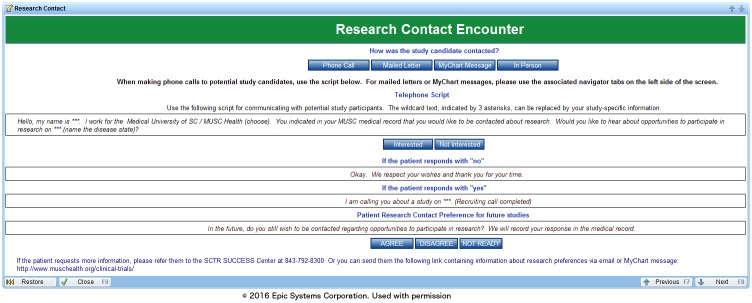
Research contact encounter.

To manage research contacts, we created a new encounter type, the “Research Contact Encounter”, and a corresponding research contact form for documentation of these contacts (Fig 2). When a research assistant contacts a potential research subject, he or she documents the mode of contact (phone call, mailed letter, patient-portal message, or in-person), if the contact is successful, and if the patient was interested in participation or further information about the research study. The last section of the contact form script asks the patient if he/she is willing to be contacted about other research opportunities. This feature was mandated by the research permissions task force because the appropriate number of contacts can only be determined by the patients themselves. With this feature, the research assistant can, based on the patient response, change research permissions directly from within the form.

### Training and access to research functionality

We created training materials for researchers and certified their competence before granting them access to Epic research functionality. Two types of courses were created: one for use of SlicerDicer for preparatory to research (feasibility) queries (included training in use of the research registry) and a second one for general use of Epic for research functionality. Separate in-person training was performed for clinic staff to demonstrate how to open the research preferences questionnaire from the nursing navigator (the place in the EHR where outpatient nurses do most of their documentation). This training was supplemented by handouts demonstrating this workflow. Printed patient handouts were created by the SCTR Regulatory Knowledge and Support Core were provided to clinic staff that describe what each of the research preference questions means, how medical information is used for research, what patients can do to change their preferences, and what information is associated with surplus tissues for research. The same material was presented on the CTSA-sponsored research site as a reference for patients accessing the questionnaire using the patient portal.

### Phase I: Capture of research preferences using the patient portal

**Fig 3:**
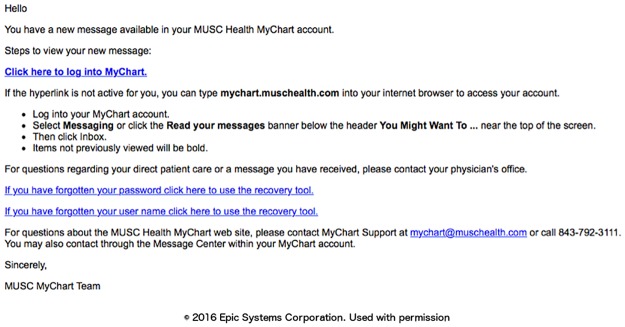
Patient portal notification email.

**Fig 4:**
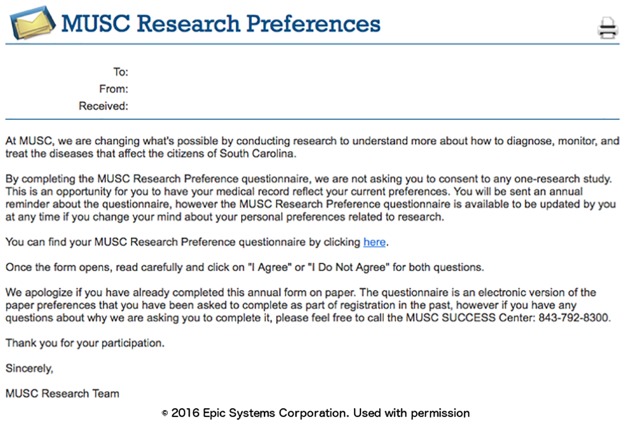
Patient portal message.

**Fig 5:**
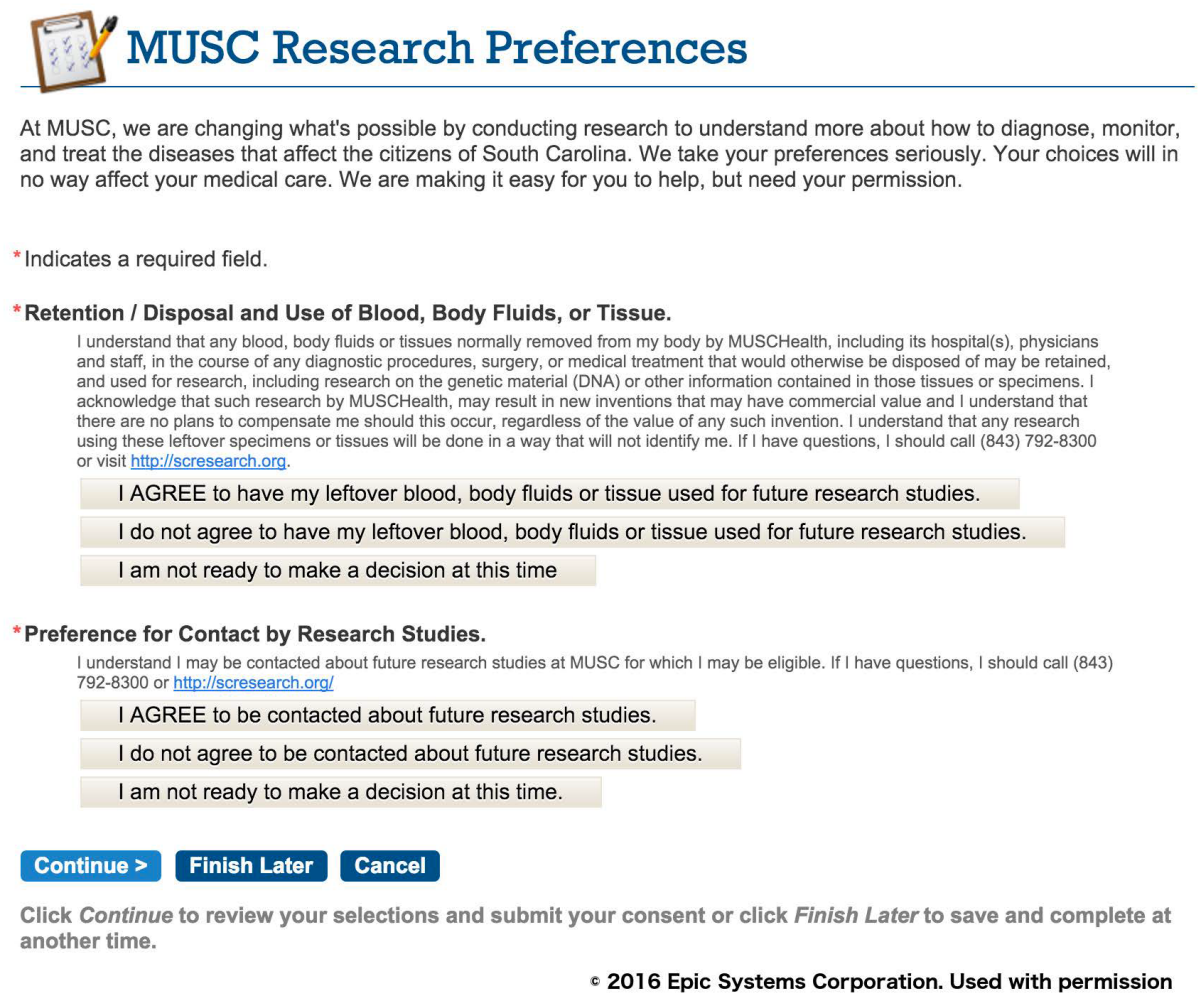
Research preferences questionnaire.

**Fig 6:**
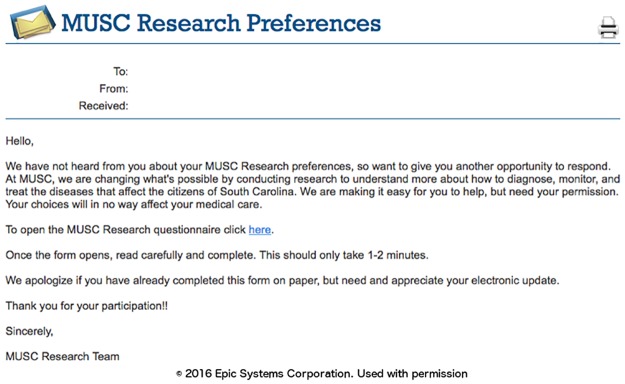
Reminder message.

**Fig 7:**
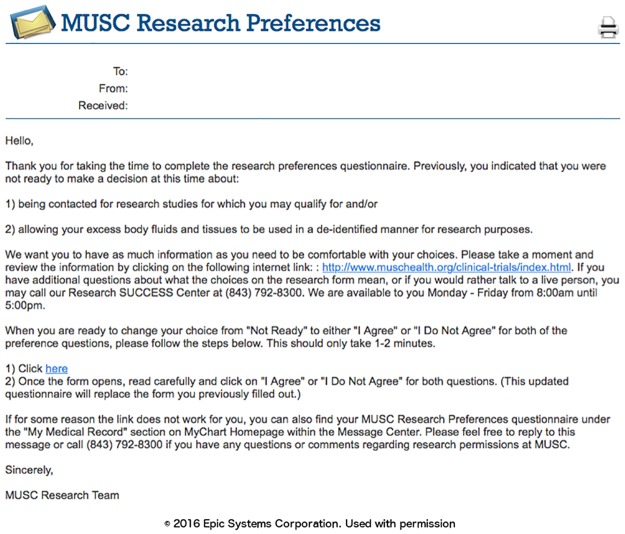
“Not ready” message.

We designed the research preferences to initially attempt to contact patients using the patient portal, as this route was the thought to be the most efficient approach to generate a large registry of patient research permissions. The first message asking the patient to declare their permission preferences was timed for at least four weeks after registration for the patient portal to allow the patient to first focus on clinical material. A portal (MyChart, Epic) message notification email was sent to all the new enrollee personal email accounts (Fig 3). Once the patient logs into the portal, they can review a message about research preferences that appears with a link to the research permissions questionnaire (Fig 4). The research preferences questionnaire contained the two questions about the patient’s preferences for both biobank and contact (Fig 5) and mirrored the prior paper process. The questionnaire was applied at a facility level. Once the questionnaire invitation was sent to the patient, they could read and review it in the questionnaire tab of the portal at their leisure. Non-respondents to the initial message receive up to two follow-up reminders (Fig 6) at one month intervals. If patients indicate the “not ready” option, they receive a message with a link to a web page designed to clarify the meaning of opting in and the general value of participation in research (Fig 7). The web page includes contact information for SCTR support staff, text explaining research permissions, and video produced by a local news station about the benefits of research. This video explains what kind of research can be available both for patients with specific conditions and for healthy volunteers. It also includes interviews with 1) a physician researcher who explains how participation today may lead to future discoveries, and 2) two volunteers that are advocates for research. Patients can also download printed materials from the web site for later review.

**Fig 8:**
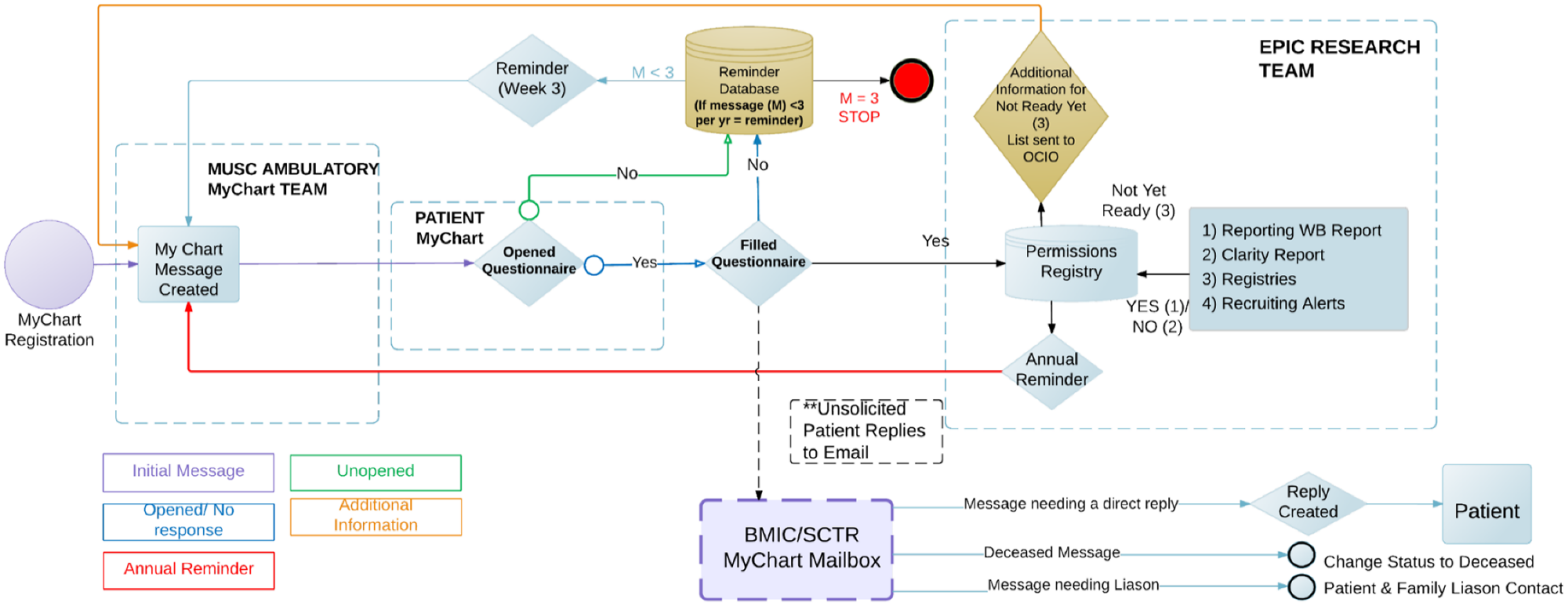
Questionnaire workflow.

All communications sent through MyChart were based on reports. Standard communications were created and sent using Epic foundation bulk communication applications at specified intervals. The overall workflow developed is shown in Fig 8.

### Phase II: In clinic capture of research preferences

**Fig 9:**
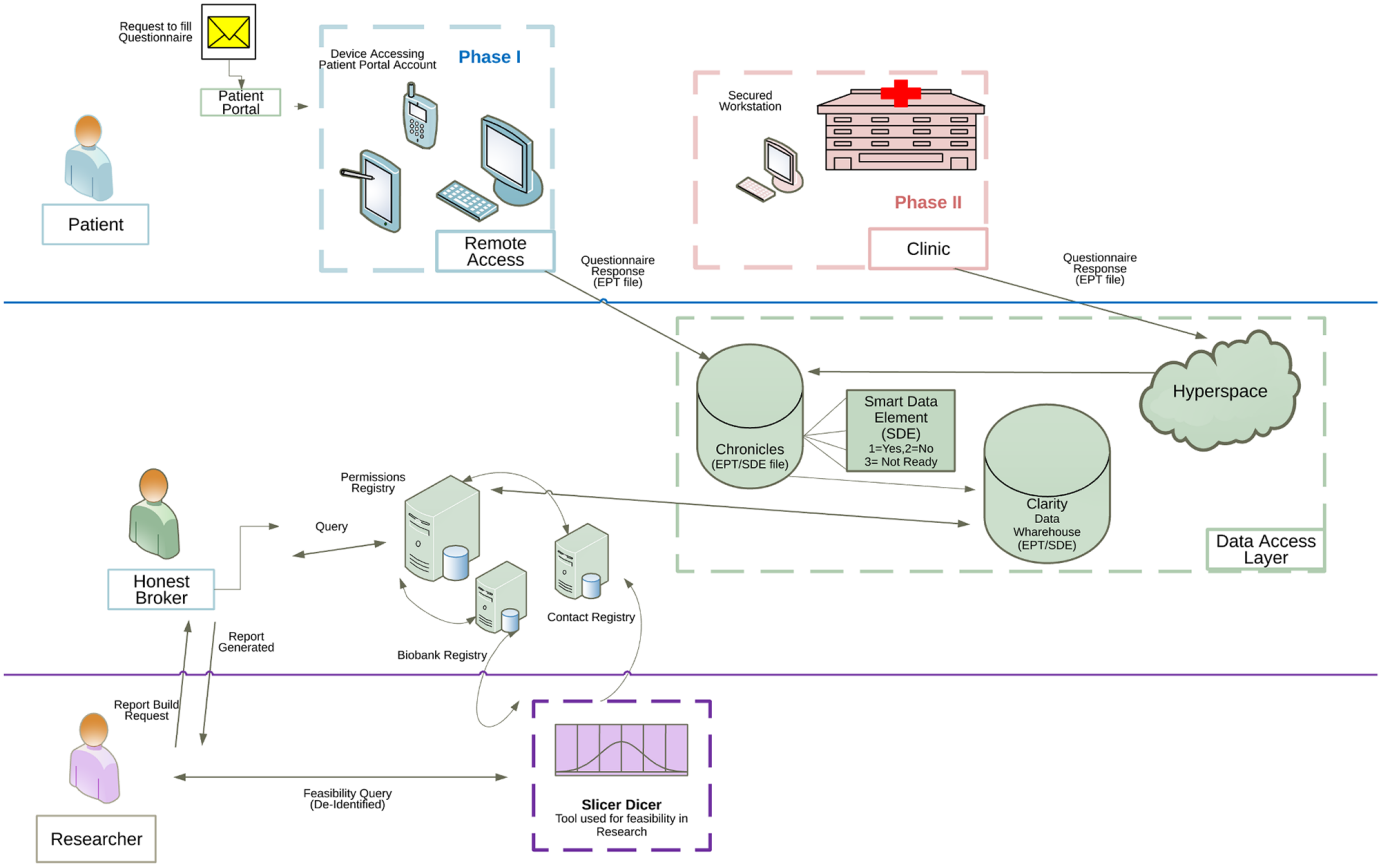
Registry process flow.

The permission collections were rolled out in phases. In Phase I, beginning on December 15, 2014, the research permissions questionnaire invitations and completions were collected exclusively using the patient portal. The data collection for this phase was completed on March 15, 2016. In Phase II, from March 14, 2016 to May 31, 2016, we captured research permissions during outpatient visits (Fig 9). Collection workflows were designed to minimize impact on clinical care and costs. We considered several solutions, including kiosks and dedicated tablet computers, that were rejected due to cost. A process was designed to capture research preferences using the exam room computer between rooming a patient and the provider visit. To implement the approach, we used the Epic captive mode questionnaire button to access to questionnaire from within visit navigator. This functionality locks the patient chart and opens the questionnaire queue. The queue shows whether the research preferences questionnaire has been completed and the last completion date. Other questionnaires, such as questionnaires for patient reported outcomes, are visible in the same queue and can be administered using the same workflow. From this screen, the patient may select and complete the research preferences questionnaire before the provider enters the room. After the patient completes the questionnaire, the terminal returns to the “secure workstation” state for rapid access to the patient’s chart by the provider. Completion of the questionnaire is optional, and the provider can close the questionnaire to avoid clinic delays. The questionnaire is always available to patients in the clinic and on the portal for those who change their minds about their research preferences.

### Initial evaluation

This project used a waiver of consent. Studies were limited to data that was deemed minimal risk and expedited by our IRB under the regulations of the Office for Human Research Protections (OHRP), Medical University of South Carolina Institutional Review Board; Research Permissions Response under approval # Pro00040823.

### Evaluation study 1: Initial mass notification of current patient portal users

This study captured the research preferences of existing MyChart users through a series of bulk email communications generated by reporting from the registry. The initial request was sent to the entire cohort of patients who were registered for a MyChart account and had been seen by a MUSC Health provider within the prior year. Two follow-up requests were sent at approximately two week intervals to non-respondents. Questionnaire responses to each mass mailing were reported as MyChart encounters by date of completion. In addition, direct email responses to the questionnaire invitation and help desk calls were also tracked. We explored the differences in the proportions of patients opting in/opting out/not ready for research contact or surplus tissue biobanking (based on their latest response) by demographic characteristics. The Chi-square test was used for testing differences, and a probability of falsely discovering differences (p value) of less than or equal to 5% was considered significant.

We then used SlicerDicer to mimic possible researcher feasibility queries to give a broad sense for the number of patients with different conditions that had opted in for contact and use of surplus tissues. Inclusion criteria for the queries were 1) inclusion in the opt-in registries and 2) Epic Diagnostic code Groupers (EDG, groupings of specific codes under more general codes) for common diagnoses.

We reported all responses longitudinally to determine the subsequent responses of patients who initially indicated that they were not ready to decide to participate. We recorded the proportion who ultimately changed their responses to opt-in or opt-out within the study period. To measure how many patients sought additional information to make their decision about research permissions, the number of page visits to the CTSA-sponsored research preferences information website (linked by the “nor ready” email) was tracked.

### Evaluation study 2: Initial implementation in clinic

Working with administrative leaders of different clinical groups, we identified a pilot site for testing implementation of in-clinic capture of research permissions. The obstetrical practice was chosen for the pilot because of ongoing needs for research patient recruitment in the practices and the a lower than average MyChart enrollment in this practice. A prototype was built in the development environment and refined with input from clinical leaders. After training of staff and key informant interviews, a modified work flow was adopted that integrated research preferences capture into an existing in-clinic workflow used for MyChart signup. Procedures were tested and refined throughout the pilot. Nurses’ opinions about the effect of the clinic capture of research permissions were obtained by in-person interviews.

Starting in March 14^th^ of 2016 and continuing to May 31, we reported the number and type of in-clinic responses using Epic Reporting Workbench. The demographics of patients completing the questionnaire in clinic were also extracted for comparison to the patient portal methods of data capture. We explored the differences in the proportions opting in by demographic characteristics, using the Chi Square test. A probability of falsely discovering differences (p value) of less than or equal to 5% was considered significant.

## Results

### Evaluation study 1

**Fig 10:**
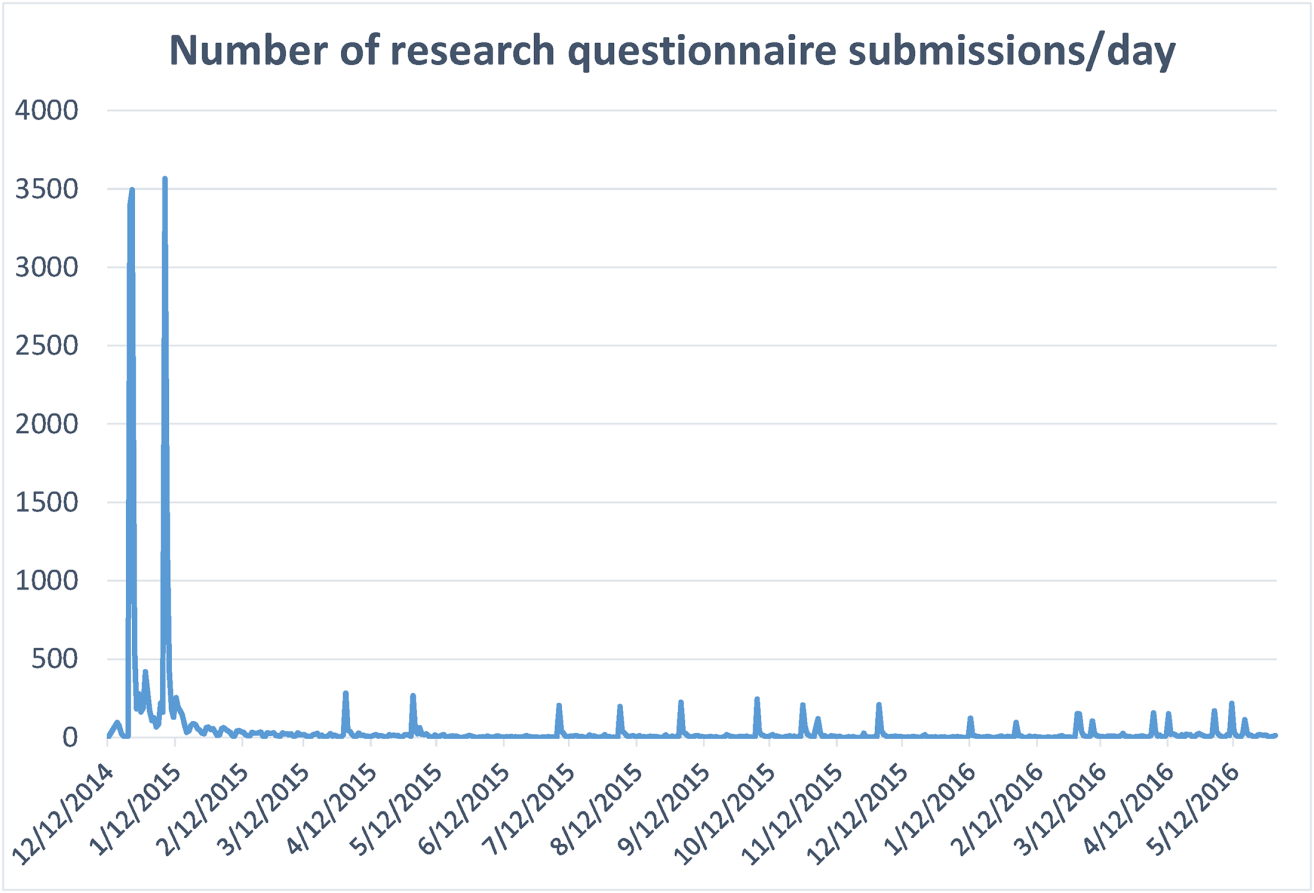
Questionnaire response over time.

In the initial phase of recruitment, messages were sent to 59,970 patients. This represented approximately 18% of all adult patients seen institution-wide. From December 15, 2014, to March 15, 2016, we received 26,679 responses to the questionnaire, corresponding to 24,247 unique patients (some having responded more than once). For each of three rounds of notifications, the response rate was greatest within the first few days and then rapidly declined (Fig 10). Sixty-five percent of responses to the request were returned within 2 days. Invitations to this questionnaire continue to be sent to new enrollees in the patient portal, and monthly bulk sends are represented as spikes in the response rate.

Among responses, the overall rate of opt-in for direct research contact was 74%. The rate of opting in for use of surplus tissues/fluids was 77%. Importantly, 10% and 10% of responses were “not ready to make a decision” about research preferences for future research contact and use of surplus tissues/fluids, respectively. There were significant differences between sex, race, and age groups with respect to these responses (Table 1). While females comprised the majority of responses to the questionnaire (resulting from a larger pool of female MyChart users and survey respondents), they were less likely opt-in (say “yes”) to research permissions questions than men. European-Americans and those older than 69 years of age were more likely to opt-in.

**Table 1:** Comparison of patient portal responses to research permissions questions by demographics.

Characteristic	Responded to Questionnaire (N = 24,247)		Yes to Biobank n = 18,671 (77%)		Yes to Contact N = 17,879 (74%)	
	n	%**	n	%**	n	%**
Gender*						
Female	15,511	64	11,584	75	11,174	72
Male	8,736	36	7,087	81	6,705	77
Race*						
European-American	20,727	86	16,548	80	15,676	76
African-American	2,609	11	1,497	57	1,601	61
Asian	183	1	116	63	106	58
Other***	728	3	510	70	496	68
Age* (Missing = 522)						
18–34	3,545	15	2,624	74	2,441	69
35–49	5,078	21	3,773	74	3,642	72
50–69	10,032	42	7,711	77	7,484	75
>69	5,070	21	4,145	82	3,948	78

Chi-square test.

* p < 0.001 for between group comparison for all factors; for both contact and biobank questions.

** Percentages rounded to the nearest integer and thus may not total 100%.

***Includes Native American, other, and unknown

The initial query captured large numbers of patients who were willing to be contacted for research with diverse medical problems. As shown in Table 2, queries using SlicerDicer returned large numbers of patients who opted in with different categories of diseases by diagnosis grouper (grouped by condition in the Epic EDG master file as “EDG Concepts”) or vital sign body mass index (BMI).

**Table 2:** Registry patients agreeing to contact/biobank per condition.

Condition	Contact (n)	Biobank (n)
EDG Concept Asthma	1,512	1,534
EDG Concept Chronic Obstructive Pulmonary Disease	512	523
EDG Concept Coronary Artery Disease	1,124	1,186
EDG Concept Depression	2,733	2,781
EDG Concept Diabetes Mellitus	2,146	2,196
EDG Concept Kidney Disease	1,762	1,844
EDG Concept Hyperlipidemia	5,329	5,558
EDG Concept Hypertension	6,739	6,965
Obesity (Body Mass Index >30)	3,645	3,724
EDG Concept Stroke	479	503

“Contact”–the patient has agreed to future contact about research

“Biobank” the patient has agreed to research use of surplus biospecimens

### Patients initially responding “not ready”

Three thousand, five hundred and ninety-five patients initially responded that they were “not ready” to decide about direct research contact or research use of surplus tissues. Within those populations 58% and 47% (biobank and contact, respectively) changed their response to “opt-in”, while 41% and 53% (biobank and contact, respectively) changed to “opt-out”. Some patients changed their preferences multiple times, and 0.4% of the patients who changed their answers from “not ready” ultimately changed their answer back to “not ready”.

### Evaluation study 2: In clinic workflow

From March 14^th^ to May 31^th^ 2016, we tracked in clinic responses to the questionnaire (Table 3). This workflow continues, and other clinics are being added in a rolling fashion. Unstructured interviews of clinic staff were performed to determine their satisfaction with the process. They reported that the process of opening the questionnaire did not hinder clinical workflow, and completion of the questionnaire did not delay patient care.

**Table 3:** Comparison of in-clinic responses to research permissions questions by demographics.

Characteristic	Responded to Questionnaire		Yes to Biobank		Yes to Contact	
	n	%**	n	%**	n	%**
Gender*						
Female	247	100	135	55	125	51
Race*						
European-American	139	58	101	73	88	63
African-American	95	37	27	28	31	33
Other***	13	5	7	54	6	46
Age* **(missing = 4)**						
18–34	152	63	69	45	71	47
35–49	63	25	46	73	39	62
50–69	26	11	14	54	12	46
>69	2	1	2	100	2	100

Chi-Square test.

* p < 0.001 for between group comparison for all factors; for both contact and biobank questions.

** Percentages rounded to the nearest integer and thus may not total 100%.

*** Includes Native American, Asian, Other, and Unknown

There were 247 female patients that responded to the survey in the obstetrics and gynecology clinic (Table 3). Overall the rates of patients opting-in for direct contact for research were lower than those seen in patient portal cohort (50.6% for research contact in clinic vs. 74% via the portal and 54.7% for biobank in the clinic vs. 77% via the portal, Tables 1 vs. 3). The rate of patients expressing that they were “not ready” to decide was 19.5% for research contact and 17.2%, for biobanking permission. Similar to with Phase I results, European-Americans and those greater than 35 years of age were more likely to opt-in; however, the observed response rate from African Americans was even lower (33% for research contact in clinic vs. 61% via the portal and 28% for biobank in the clinic vs. 57% via the portal, Tables 1 vs. 3).

## Discussion

The requirement for opt-in consent for tissue use is at the forefront of debate about proposed changes to the Common Rule [7, 8]. The results of our study suggest that, by the number of preferences recorded, it is feasible to take an institution from an opt-out to an opt-in approach using EHR-based questionnaires in the patient portal and in-clinic workflows. Prior work suggests patients strongly want to be asked about research participation, and the opt-in approach is one way to give patients choice [29]. The opt-in policy is not currently required and can certainly have weaknesses [30, 31]. However, its adoption may further protect the rights of human subjects in light of rapid evolutions in genomic and other technologies.

Our approach to opt-in consent is not strictly compliant with the Notice of Proposed Rule Making (NPRM) [7] and is more akin to obtaining *permission* than the *broad-consent* strategies discussed in the NPRM. In particular, our approach does fully discuss the risks and benefits of research and does not use a “handwritten signature” to capture consent. Rather, it uses authentication methods in the EHR to verify identity. We speculate that the three questionnaire responses offered gives patients more options to express their preferences than the prior passive opt-in approach. However, it is not informed consent. Thus while our results speak to the feasibility of an active opt-in approach, achieving the ambitious goals of the NPRM would require further modifications.

In our patient portal sample, response rate to our permissions survey was 35%. This response rate suggests that, even among portal users, alternative methods to capture responses for research permissions are necessary. The overall opt-in rate for research contact participation was about 25% of the total population queried. Although difficult to generalize from specific survey response rates to general research permissions, this rate is comparable to the response rates in a case study of using a patient portal to recruit research participants for a survey study [32]. In that study, 16% of those receiving a portal invitation completed a survey. Another study that compared response rates for surveys using postal, internet, and telephone modes found that the highest response rate was telephone contact of volunteers (30%), while internet survey responses were low, at 4.7% [33]. Response rates for recruitment for social network communities in a 2014 systematic review ranged from 2% to 27% [34]. We speculate that our response rate may, in part, be due to several factors: 1) implementation of the survey within the familiar environment used for clinical communications, 2) increased patient control over permissions through the “not ready” to decide option and the ability modify preferences at any time via the portal, 3) the brevity of the questionnaire, and 4) giving patients both live and web-based support to answer patients’ questions about research and research permissions. However, the effect of each of these factors on response rates was not directly addressed by this study.

While those responding may not represent all portal users, the results here challenge the assumption that opt-in approach will significantly reduce patient participation. Seventy-two percent of patients opted-in for direct contact for research, and 75% of patients opted-in for use of biospecimens via MyChart. This is somewhat similar to the 77% passive opt-in rates for contact in our prior study [25–27]. Among African Americans, the opt-in rate was lower, with only 57% opting in for surplus tissue usage, and 61% opting in for research contact. The reasons for this lower response rate were not explored in this study. These results support the notion that a patient-centric approach that is integrated into the EHR patient portal and clinic workflows may be practical at other institutions as well as ours.

Our questionnaire of research permissions included a “not ready” choice. We found that substantial percentages of patients (10–11%) indicated that they were “not ready” to decide about research contact and use of surplus biospecimens. Additional work is needed to explore how best to educate patients about the rationale and options for research participation. It was unclear if those who indicated that they are “not ready” wished to learn more about research permissions before deciding, were truly uncertain about the concept of participation, or were politely opting out. However, in our population, about half of those who initially indicated they were “not ready” changed their responses to “opt-in”.

A limitation of our initial preference capture methodology was that it only reached patients enrolled in our institution’s patient portal. As shown in our results and in prior work, portal users are a demographically distinct group. [35] In the second phase, we examined recruitment rates in clinic. In this early pilot study, rates of opting in for contact were lower than portal responses. It is notable that the pilot clinic location included many patients that were pregnant. However, further work is needed to assess whether this lower response rate will continue as we implement in-clinic data capture in other clinics.

While our approach to capturing research permissions now includes workflows for our patient portal and for outpatient clinics, many patients’ first and only contact with our institution is either in the emergency department or through an inpatient admission. Work is underway to develop approaches to capture research permissions in these other contexts.

Our approach used standard workflows for clinical communications. This required close collaboration with our institution’s information technology leadership and with clinical leaders. It resulted in compromises such as prioritization of clinical over research and the one month delay of research questionnaire release after portal registration. Other researchers have described offering research contact permissions questionnaires when patients first discover patient portal functions [15].

There are also disease- or condition-specific registries that have demonstrated that “permission to contact” increases the efficiency and success rates of enrollment [22]. Many existing research contact registries rely upon the patient to define their disease or health status [15, 16, 18]. Some registries also include patients’ preferences for the types of studies in which they want to participate [15, 16, 18]. In our study, a few inquiries about disease-specific research were received from patients responding to the questionnaire. The approach of modeling research participation preferences by disease increases the complexity of preference questionnaires and the data structures for recording of research permissions in the patient’s record. However, currently, there is little evidence that shows that allowing patients to specify the types of studies they want to be notified about improves enrollment rates, and this issue deserves further study. However, others have noted that engagement of participants in biobank registries might increase enrollment. For example, if regular updates about the biobank were provided, this might increase patient participation [2].

In Table 4, we summarize the features of several ongoing programs to create registries of potential research volunteers. This review suggested that our approach may be one of the first population-based efforts in which patient preferences and EHR data elements can be queried from within the EHR.

**Table 4:** Comparison of research recruitment registry characteristics from information available on public sources.

Research Registry	Description	Registry Inclusion	Phenotyping	Contact	Biobank
**Medical University of South Carolina**	Population based, single institution	Population based, within EHR	EHR defined	Yes	Yes
**University of Pittsburg** (http://www.researchregistry.pitt.edu) [19]	Multi-institutional	Self-defined but some inclusion from participating clinical outpatient offices and MyUPMC	Patient defined	Yes	No
**University of Kansas Medical Center (KUMC) and other regional hospitals and universities** (https://pioneersresearch.org/) [16]	Multi-institutional	Self-defined, and community engagement efforts	Patient defined and, when possible, also connects EHR to participants.	Yes	No
**ResearchMatch** (http://www.researchmatch.org) [14]	Institutionally independent, national	Self-defined	Patient defined	Yes	No

By integrating the research permissions registries in the EHR, researchers can use a broad range of discreet EHR data as inclusion rules for feasibility and recruiting reports. An important advance, which greatly enhances the usefulness of the EHR for this task, is the integration of SlicerDicer self-service queries into the researcher permissions. However, there are still limitations to this approach. Defining complex temporal phenotypes may require data export [36]. Much of the clinical data in EHRs is still in free text form. Natural language processing tools may also be helpful to define phenotypes and identify patients eligible for clinical trials [37–39].

## Conclusions

This project demonstrates the successful initial use of an EHR system to convert an institution from an opt-out to and opt-in approach for obtaining preference for participation in research. Reductions in the number of patients potentially available from the initial institution-wide passive opt-in approach were at least partially offset by the ease of access to permissions data in the EHR. Access to standard query and reporting applications from within the EHR enables rapid generation of feasibility and recruiting reports. Additionally, by integration of research permissions registries in the EHR, researchers can leverage existing EHR population health management functions to efficiently contact patients for participation in research studies. Capturing consent requires use of both portal-based and in-clinic methods to reach a majority of patients in a population; though, large registries can be accrued quickly with just portal questionnaires. Because of the large proportion of patients who prefer not to participate in research, an opt-in consent appears to be an essential part of patient-centric approach to conducting clinical and translational research, particularly among African-Americans, at our institution.

## Supporting information

S1 Fig Research preferences flyer. (PDF)

S2 Fig Research preferences questionnaire. (PDF)

S1 Table MyChart preferences basic demographic dataset (date shifted). (XLSX)

S2 Table In-clinic preferences basic demographic dataset (date shifted). (XLSX)
