# Supplementary material for: A population-based approach for implementing change from opt-out to opt-in research permissions
Source: PLoS One. 2017 Apr 25;12(4):e0168223. doi: 10.1371/journal.pone.0168223 (PMC5404843; doi:10.1371/journal.pone.0168223)
Supplement: S1 Fig — (PDF) [file pone.0168223.s001.pdf]

YOUR SECOND QUESTION:
Contact for Future Research

You may be interested in research studies by Researchers who are not involved with your regular medical care. Please tell us your preference for being contacted to learn about these future research opportunities.

If you agree, you could be contacted a number of different ways such as through you your MyChart account, or by email, phone or letter.

If you say no, your doctors can still suggest studies that he/she feels may assist with your clinical care.

Participating in any research study is voluntary and just because you may agree to be contacted it in no way means that you have to participate in a study. It is your choice to participate or not, any time you are invited.

Log into your MyChart questionnaire online and let MUSC know your choice by selecting the option that's right for you.

An example of you choices will be:

I AGREE...
I do not agree...
I am not ready to make a decision at this

Do not mark this brochure. You can only let us know your preference online at https://mychart.muschealth.com/mychart/

What if I Change My Mind?

At any time, you can access the Research Preferences Questionnaire in MyChart and change your preferences.

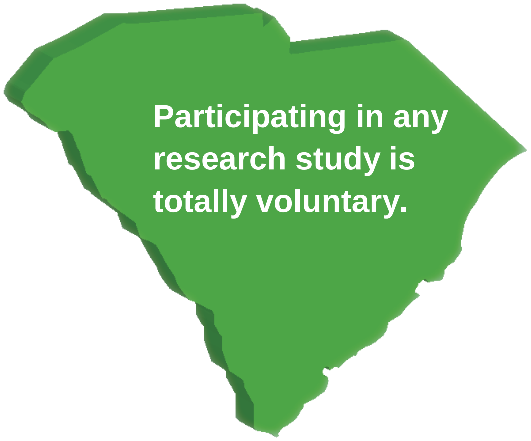

For further information about Research Permissions, please contact the SCTR SUCCESS Center at MUSC.

SUCCESS CENTER
SCTR Research Support Services

Phone: 832-792-8300

Email: Success@musc.edu

Or Visit the MUSC Health Clinical Trails webpage

http://www.muschealth.org/clinical-trials/

MUSC

Information about MUSC Research Preferences Questionnaire

You are being asked to choose your preference about two research related items found in MyChart. This brochure will explain what the two items listed on your Research Preferences Questionnaire mean and who you can contact if you have questions or need more information.

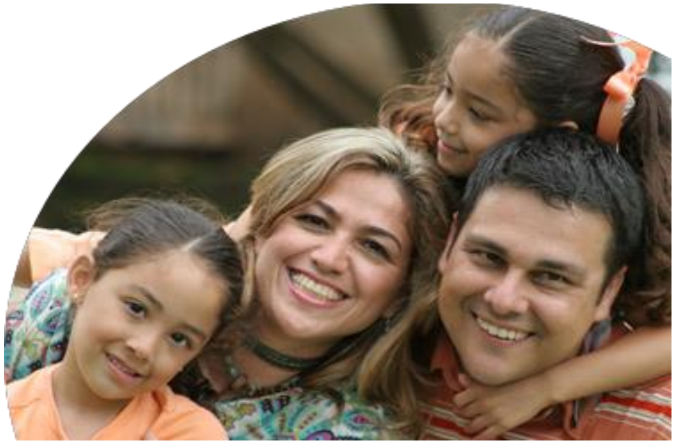

Research is important for cures!

YOUR FIRST QUESTION:

Retention / Disposal and Use of Blood, Body Fluids, or Tissue

As part of your regular medical care, samples of your blood and/or body fluids may be collected, tested and results sent to your doctor. If you agree, any unused samples may be used for research. Normally, these remaining samples would have been discarded (thrown away).

As part of your regular medical care, tissue samples may be removed during surgery, then tested and results sent to your doctor. If you agree, only tissue that is not needed for testing may be used for research.

We will not collect any extra samples. Nothing will be done that is not part of your regular medical care.

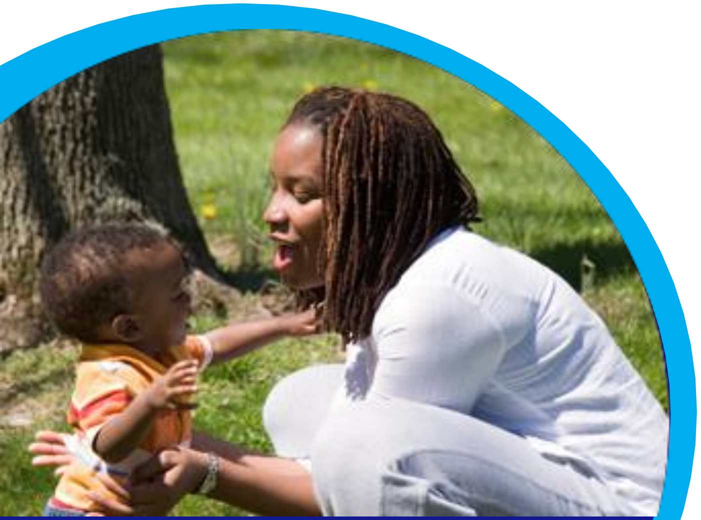

Your choices will not change the care you would normally receive.

Your Medical Information:

Along with your samples, non-identifiable information from your records at MUSC may also be used for research. Researchers who use this are trained to protect such information.

All research is reviewed by a review board (group of experts in research and ethics) that protects people in research. Your samples, diagnoses or other medical information could be used by researchers, but **they will NOT be given your name or anything else that could identify you.**

Results from any tests performed on your samples will not be included in your medical record because researchers will not know to whom the samples belong.

Genetic Research:

DNA is found in the genes, and genes are important because they are the blueprints used to build healthy bodies. Some research may involve studies of DNA to see if they contain changes that may be passed from parents to children.

These studies might help to identify changes that may lead to cancer or other diseases. DNA studies are important for finding and treating certain diseases.

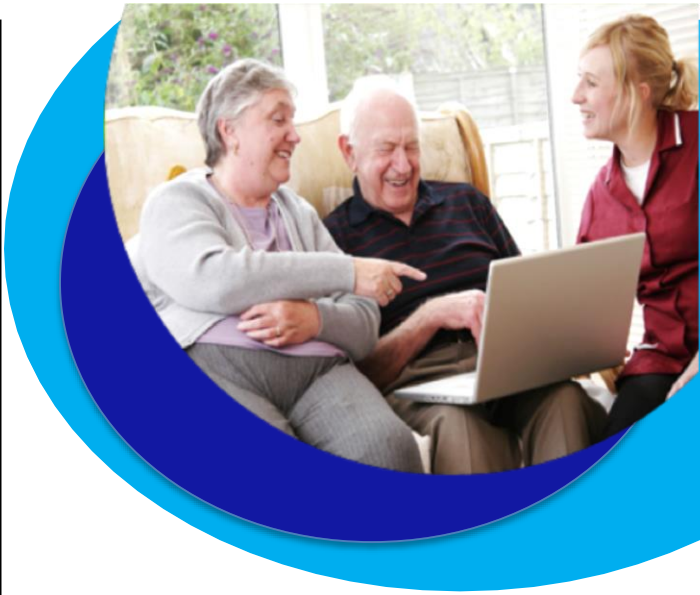

You will not know if your samples are ever used for research. You will not benefit financially if discoveries are made using your samples.

Log into your MyChart questionnaire online and let MUSC know your choice by selecting the option that's right for you.

An example of you choices will be:

I AGREE…
I do not agree…
I am not ready to make a decision at this

Do not mark this brochure. You can only let us know your preference online at https://mychart.muschealth.com/mychart/
