# Supplementary material for: A population-based approach for implementing change from opt-out to opt-in research permissions
Source: PLoS One. 2017 Apr 25;12(4):e0168223. doi: 10.1371/journal.pone.0168223 (PMC5404843; doi:10.1371/journal.pone.0168223)
Supplement: S2 Fig — (PDF) [file pone.0168223.s002.pdf]

MUSC Research Preferences

At MUSC, we are changing what's possible by conducting research to understand more about how to diagnose, monitor, and treat the diseases that affect the citizens of South Carolina. We take your preferences seriously. Your choices will in no way affect your medical care. We are making it easy for you to help, but need your permission.

Indicates a required field.

Retention / Disposal and Use of Blood, Body Fluids, or Tissue.

I understand that any blood, body fluids or tissues normally removed from my body by MUSCHealth, including its hospital(s), physicians and staff, in the course of any diagnostic procedures, surgery, or medical treatment that would otherwise be disposed of may be retained, and used for research, including research on the genetic material (DNA) or other information contained in those tissues or specimens. I acknowledge that such research by MUSCHealth, may result in new inventions that may have commercial value and I understand that there are no plans to compensate me should this occur, regardless of the value of any such invention. I understand that any research using these leftover specimens or tissues will be done in a way that will not identify me. If I have questions, I should call (843) 792-8300 or visit http://www.muschealth.org/clinical-trials/.

I AGREE to have my leftover blood, body fluids or tissue used for future research studies.

I do not agree to have my leftover blood, body fluids or tissue used for future research studies.

I am not ready to make a decision at this time

Preference for Contact by Research Studies.

I understand I may be contacted about future research studies at MUSC for which I may be eligible. If I have questions, I should call (843) 792-8300 or http://www.muschealth.org/clinical-trials/

I AGREE to be contacted about future research studies.

I do not agree to be contacted about future research studies.

I am not ready to make a decision at this time.

Continue **Finish Later** **Cancel**

Click *Continue* to review your selections and submit your consent or click *Finish Later* to save and complete at another time.

If at any time you would like to update your research preferences, you may modify them from the Questionnaires section in MyChart.

Thank you very much for your interest in research at MUSC.
